# Measuring the healthfulness of food retail stores: variations by store type and neighbourhood deprivation

**DOI:** 10.1186/1479-5868-11-69

**Published:** 2014-05-23

**Authors:** Christina Black, Georgia Ntani, Hazel Inskip, Cyrus Cooper, Steven Cummins, Graham Moon, Janis Baird

**Affiliations:** 1 Medical Research Council Lifecourse Epidemiology Unit, University of Southampton, Southampton General Hospital Tremona Road, Southampton SO16 6YD, United Kingdom; 2 NIHR Nutrition Biomedical Research Centre, University of Southampton, Southampton SO16 6YD, United Kingdom; 3 Department of Social and Environmental Health Research, Faculty of Public Health & Policy, London School of Hygiene & Tropical Medicine, 15-17 Tavistock Place, London WC9H 1SH, United Kingdom; 4 Geography and Environment, University of Southampton, University Road, Southampton SO17 1BJ, United Kingdom

**Keywords:** Food environment, Consumer nutrition environment, Diet quality, Dietary inequalities

## Abstract

**Background:**

The consumer nutrition environment has been conceptualised as in-store environmental factors that influence food shopping habits. More healthful in-store environments could be characterised as those which promote healthful food choices such as selling good quality healthy foods or placing them in prominent locations to prompt purchasing. Research measuring the full-range of in-store environmental factors concurrently is limited.

**Purpose:**

To develop a summary score of ‘healthfulness’ composed of nine in-store factors that influence food shopping behaviour, and to assess this score by store type and neighbourhood deprivation.

**Methods:**

A cross-sectional survey of 601 retail food stores, including supermarkets, grocery stores and convenience stores, was completed in Hampshire, United Kingdom between July 2010 and June 2011. The survey measured nine variables (variety, price, quality, promotions, shelf placement, store placement, nutrition information, healthier alternatives and single fruit sale) to assess the healthfulness of retail food stores on seven healthy and five less healthy foods that are markers of diet quality. Four steps were completed to create nine individual variable scores and another three to create an overall score of healthfulness for each store.

**Results:**

Analysis of variance showed strong evidence of a difference in overall healthfulness by store type (p < 0.001). Large and premium supermarkets offered the most healthful shopping environments for consumers. Discount supermarkets, ‘world’, convenience and petrol stores offered less healthful environments to consumers however there was variation across the healthfulness spectrum. No relationship between overall healthfulness and neighbourhood deprivation was observed (p = 0.1).

**Conclusions:**

A new composite measure of nine variables that can influence food choices was developed to provide an overall assessment of the healthfulness of retail food stores. This composite score could be useful in future research to measure the relationship between main food store and quality of diet, and to evaluate the effects of multi-component food environment interventions.

## Background

There is increasing evidence that the food environment is an important determinant of dietary behaviour and obesity [1,2]. With obesity accounting for almost 21% of health care costs in the US [3] and the UK’s NHS spending more than £5 billion a year on obesity-related health problems [4], governments are exploring policy options that modify the food environment to make healthier choices easier for consumers [5,6].

Glanz et al. [7] have developed a conceptual model to guide food environment research. The focal points of the model are the four types of food environments: *community nutrition environment*, *consumer nutrition environment*, *organisational nutrition environment* and *information environment*. The majority of food environment research has focused on the community nutrition environment [1], which measures the number, type, location and accessibility of food sources [7]. Fewer studies have assessed the consumer nutrition environment [8] which considers factors that influence food choice within stores such as availability, price, promotions, placement, variety, quality and nutrition information [7].

Assessment of the consumer nutrition environment in retail food stores is important because of the global convergence of shopping habits away from smaller specialty stores towards stores that stock a wider range of products [9]. Consumer’s dietary choices are affected by the products sold, prices charged and promotional strategies used in their main food stores [10]. More healthful food store environments could be defined as those which promote healthful food choices such as selling good quality healthy foods or placing them in prominent locations to prompt purchasing.

A number of tools to assess the consumer nutrition environment have been developed [11,12], with the vast majority for use in the United States (US). Few tools have undergone reliability or validity testing or provide the level of detail required to assess linkages between retail food environments and dietary behaviour [11,13,14]. A great proportion of tools measure two in-store factors: availability and price [11,12,15]. A smaller number of tools have assessed variety and/or quality of fruit and vegetables [16-18], in-store advertising and/or product placement [13,19,20] or price promotions and nutrition labelling [21].

Some tools enable the creation of a composite score of the in-store environment including the widely used Nutrition Environment Measures Survey–Stores (NEMS-S) [17] and the *CX*^*3*^ Food Availability and Marketing Survey [20]; both developed for the US context. The NEMS-S scores and *CX*^*3*^ store scores incorporate three in-store factors: availability of healthier products, fruit and vegetable quality, and price or in-store advertising. The Health Responsibility Index was developed to measure the in-store environment of nine supermarkets in the UK [21]. It measured sodium content, nutrition labelling and information, and price promotions on twelve frequently consumed processed products known to be high in sodium. Composite scores incorporating several consumer nutrition environment factors can provide an overall evaluation of the store environment and have been shown to help communities and policy makers in the US identify priority areas and inform interventions [20]. However, no score has included more than three in-store factors or included standardised measures that can be used to statistically assess relationships between diet and in-store environments or monitor relative change in environment over time.

There is a gap in the literature for a comprehensive tool that measures multiple in-store factors concurrently on healthy and less healthy products, particularly outside the US. Such a tool could provide a thorough evaluation of differences in the retail food store environment by store type and neighbourhood deprivation and may identify target sites for interventions.

In the literature, supermarkets are portrayed as offering the healthiest shopping environment for consumers and small convenience stores the poorest [22,23]. These broad categories however, cover a heterogeneous group of stores [10,24]. In the UK for example, there are four different types of supermarkets that target different consumer groups [9,10] and are likely to offer different shopping environments. Research that excludes the full range of environmental exposures or measures only healthy or less healthy foods may be misrepresenting the food environment within these stores.

Area based differences in the consumer nutrition environment have been observed in the US but no clear trend has been seen in other high income countries [25,26]. In-store assessments based on a limited number of environmental factors and foods may be missing important socio-economic differences. An observation tool that evaluates several environmental exposures of foods commonly used to assess dietary disparities may provide a more complete environmental assessment.

This study addresses a current gap in the literature by developing a comprehensive consumer nutrition environment observational tool to measure the ‘healthfulness’ of food retail stores in the UK and testing differences by store type and neighbourhood deprivation.

## Methods

### Consumer nutrition environment tool development

A list of all retail food stores and their postcodes in six council boundaries (Southampton, Eastleigh, Fareham, Gosport, Havant, Portsmouth) within Hampshire, UK, was compiled in July and August 2010. Store information was obtained from council Food Safety Registers and on-line business directories (yellow-pages and yell.com). Between July 2010 and June 2011 trained fieldworkers ‘ground-truthed’ the study area and collected data in 601 of the 606 retail food stores.

A consumer nutrition environment tool was designed to measure nine factors that can affect consumer’s food choices. Data on number of *varieties*, *price*, *promotion*, *shelf placement* and *store placement* were collected on seven healthy and five less healthy products. In addition, information on the type of *nutrition information* and availability of a *healthier alternative* were collected for less healthy products. The *quality* of two fruits and four vegetables and opportunity for *single sale* of the two fruits were also measured. Table 1 describes the definitions and measurement scales of the variables included in the tool. Information on fruit and vegetable quality was collected using a published quality indicator [18]. Data on the remaining variables were collected using novel measures. The tool and survey protocol are available in the Additional file 1. The median time taken to complete the survey across the 601 stores was 11 minutes (IQR: 7, 15).

**Table 1:** Variables measured in the consumer nutrition environment tool

	**Variable**	**Definition**	**Measurement scale**
I	Variety	*The number of different choices within a product range based on: product flavour, product size, fair trade/ organic range or no-name/low-cost range*	*Not available, 1, 2, 3, 4, 5+*
II	Price	*Price of the cheapest item, £ per portion, for each product*	*Pound sterling per portion*
III	Promotions	*Whether or not the product category was on price promotion*	*Yes/ no*
IV	Shelf placement	*Where on the shelf the cheapest item for each product was placed*	*Bottom shelf, other, prominent (eye-level)*
V	Store placement	*Which part of the store the cheapest item for each product was placed*	*Inconspicuous, noticeable, prominent*
VI	Quality	*Level of quality of the two fruit and four vegetables*	*Poor, medium, good *[18]
VII	Healthier alternative	*Whether or not a healthier option was available for less healthy products*	*Yes/ no*
VIII	Nutrition information	*The type of nutrition information available on the cheapest item for each product*	*None, other*^a^*, back-of-pack, front & back of pack*
IX	Single fruit sale	*Whether or not single sale of the two fruit measured was possible*	*Yes/ no*

^a^ For example recipe card.

The 12 food products were: peppers, tomatoes, lettuce, onions, apples, bananas, wholemeal bread, oven chips, sausages, crisps, sugar and white bread. Products were selected because they discriminate between better or poorer dietary patterns, are frequently consumed in England [27] and could be measured in a large survey. The food products selected represent items from short and long food frequency questionnaires (FFQ) used to determine differences in dietary quality among a number of populations including young women, young children and older adults [28-31]. These foods represent the UK Department of Health’s dietary recommendations and foods known to contribute to nutrition-related chronic diseases [29].

The level of agreement between fieldworkers was assessed by the Kappa statistic on a sample of 14 stores (large supermarket (n = 2), discount supermarket (n = 1), small supermarket (n = 4), ‘world’ store (n = 1), convenience store (n = 5), petrol store (n = 1)). The relative consistency of *price* responses was assessed using the coefficient of variation: the standard deviation of difference divided by the mean (%). Cronbach’s alpha was used to assess the internal consistency of all nine components of the healthfulness score.

### Healthfulness score development

**Figure 1:**
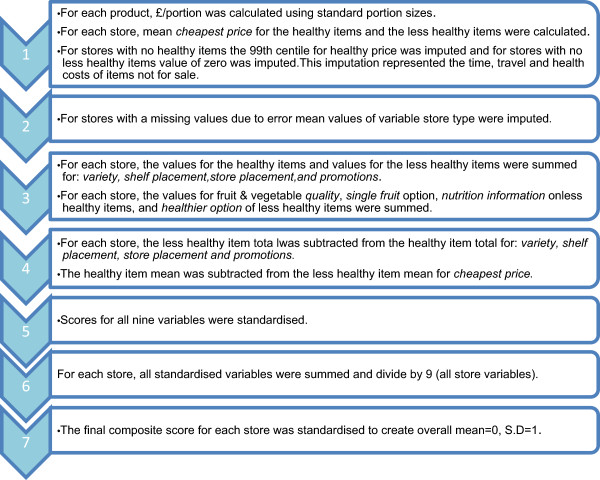
Process to create a composite score of healthfulness for retail food outlets.

In 2012, a composite score of healthfulness was created for each store surveyed, where each of the nine in-store variables were weighted equally. Seven steps were taken to create the healthfulness scores (Figure 1). Individual scores for each of the nine variables were calculated using steps one to four. All scores were constructed such that higher scores represented more healthful environments. Principal components analysis was applied in an attempt to weight the nine variables however no interpretable patterns were identified.

The process of creating the scores involved: i) converting *price* measures to pound per portion (using standard portion sizes [32]) and for each store, subtracting the mean healthy item price from the mean less healthy item price, ii), imputing missing values, due to field work error, using the mean value for the variable of that store type, iii) creating summed scores for each store for *quality* of fruit and vegetables*, single fruit sale, nutrition information* on and *healthier alternative* of less healthy products, iv) creating a score for *variety, shelf placement, store placement* and *promotions* for each store by calculating the difference between the sum of less healthy scores and the sum of the healthy scores, v) standardising all scores vi) creating a composite score for each store by summing the nine standardised variable scores and dividing by nine, vii) standardising the healthfulness scores (sample mean = 0, SD = 1).

For stores which sold no healthy items the rounded 99^th^ centile of healthy items was imputed as the mean healthy price score. This value represented the time, travel or health costs consumers could bear for healthy products not being available. Stores with no less healthy items were given a mean less healthy price score of zero. Overall, less than 1% of score components were imputed.

### Predictors of healthfulness – store type and neighbourhood deprivation

Stores were classified into seven categories based on a combination of the Local Authority Enforcement Monitoring System (LAEMS) [33] and previous UK research [34] (Table 2). Store categorisation was confirmed during data collection. A box plot was created to examine the spread of healthfulness scores across the seven store types. To assess differences in store healthfulness and individual in-store factors by store type, analysis of variance was used for normally distributed variables, Kruskal Wallis test for non-parametric variables, and Chi squared test was used for categorical variables.

**Table 2:** Retail food outlet categorisation system

**Code**	**Store type**	**Description**	**Examples**
0	Premium supermarket	5+ manned cash registers	Waitrose, M&S
		Promoted as offering highest quality goods and service	
1	Large supermarket	5+ manned cash registers	Tesco, Sainsburys, Asda, Morrisons
		All foods & many varieties	
		Majority of supermarket share	
2	Discount supermarket	5+ manned cash registers	Aldi, Lidl, Iceland, Netto, Kwiksave
		Heavily promoted as low price stores	
3	Small supermarket	1-4 manned cash registers	Tesco express, Co-op, Sainsburys local
		Smaller store of known brand name	
4	‘World’ store	1-4 manned cash registers	Asian supermarkets, Polish supermarkets, World foods
		Products for specific ethnicities	
5	Convenience store	1-4 manned cash registers	Spar, OneStop, MACE, Independent stores
		Limited number of products	
		Independents & ‘symbols’^a^	
6	Petrol station store	Sell petrol/diesel	Shell Select, Tesco petrol station, BP, M&S
		Includes small supermarkets that sell petrol	

^a^ ‘Symbol’ convenience stores are affiliated with a symbol group brand such as OneStop and Spar.

Neighbourhood deprivation was measured using the 2007 English Index of Deprivation (ID) income domain. The index of multiple deprivation (IMD) was not applied because of circularity with the access to services domain which included an access to grocery stores measure. The ID is available for Lower Super Output Areas (LSOA), small areas constructed from the 2001 English census that are socially homogenous and have a population size between 1000–1500 residents [35]. Each LSOA in the study area (n = 550) was assigned to a quintile of deprivation using the national ranks of 2007 ID income domain [35] (1 = most deprived and 5 = least deprived). Test for trend was performed to examine differences in store healthfulness and individual in-store variables by neighbourhood deprivation. Differences by LSOA rural and urban classification were not assessed because more than 98% of the study area was classified as urban [36] leaving inadequate variability.

## Results

The response rate for retail food stores in the study was 99% (n = 601). Four convenience and two ‘world’ stores refused to take part in the study. Table 3 presents the sample by store type and neighbourhood deprivation quintile. The greatest proportion of stores was convenience stores (45%, n = 268), followed by small supermarkets (21%, n = 127); large supermarkets made up 5% (n = 32) of the sample. Most retail food stores were located in the second most deprived and middle deprivation neighbourhoods (26%, n = 154 and 28%, n = 171 respectively).

**Table 3:** Store sample by store type and level of neighbourhood deprivation

**Store type**	**Most deprived**	**2**	**3**	**4**	**Least deprived**	**Total**	**(%)**
						**n**	
Premium supermarket	1	5	2	2	0	10	(2)
Large supermarket	7	9	8	4	4	32	(5)
Discount supermarket	8	7	12	3	5	35	(6)
Small supermarket	13	31	34	24	25	127	(21)
‘World’ store	17	20	16	6	2	61	(10)
Convenience store	44	72	77	39	36	268	(45)
Petrol station store	10	10	22	8	18	68	(11)
Total n	100	154	171	86	90	601	
(%)	(17)	(26)	(28)	(14)	(15)		(100)

Inter-rater reliability revealed almost perfect agreement for *single fruit sale*, *healthier alternative* and *nutrition information*, *variety and promotions* (kappa ≥ 0.85) [12]. The inter-rater reliability for *store placement* and *shelf placement* showed substantial agreement (kappa ≥ 0.73). However, *quality* of fruit and vegetables showed moderate agreement between field workers (kappa = 0.60). The coefficient of variation observed for *price* was 17%, which showed little variation in price between field workers across all products. The Cronbach’s alpha for the standardised components of the healthfulness score was 0.86.

### Predictors of healthfulness score

**Figure 2:**
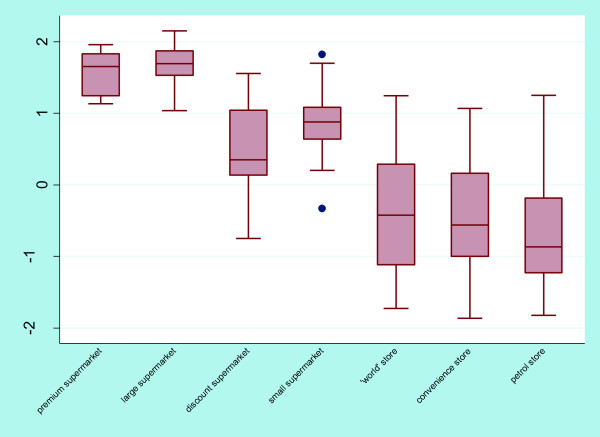
Box and whisker plot of store healthfulness by store type.

Figure 2 indicates that the healthfulness scores were poorer for ‘world’, convenience, and petrol stores. Discount supermarkets had the lowest median score of all supermarkets (Table 4) and showed the greatest spread of healthfulness scores for supermarkets. Healthfulness scores were highly positive for premium and large supermarkets, indicating that these stores offered the most healthful environments for consumers. Small supermarkets showed more variation in healthfulness scores than premium and large supermarkets, though scores remained predominantly above zero suggesting better than average healthfulness. Discount supermarkets, ‘world’, convenience and petrol stores all showed a varied distribution. ANOVA revealed evidence of a difference in healthfulness according to store type (p < 0.001). Store type explained 53% of the variance of healthfulness. Adding neighbourhood deprivation quintiles to the model did not change the variance explained. Table 4 shows that the nine individual variables followed similar trends to the composite score across the seven different store types.

**Table 4:** Individual variable and composite scores by store type

***Variable***	**Premium supermarket**	**Large supermarket**	**Discount supermarket**	**Small supermarket**	**‘World’ store**	**Convenience store**	**Petrol store**	***Possible range***^***f***^		
	***Median (IQR)***^***d***^							***Min***	***Max***	***p-value***
**Composite score**	1.7	1.7	0.3	0.9	-.04	-0.6	-0.9	*-1.9*	*2.2*	<0.001^a^
	(1.2 to 1.8)	(1.5 to 1.9)	(0.1 to 1.0)	(0.6 to 1.1)	(-1.1 to 0.3)	(-1.0 to 0.2)	(-1.2 to -0.2)			
Variety	8	8	-8	-6	-2	-9	-7	*-25*	*35*	<0.001^a^
	(7 to 8)	(6 to 9)	(-10 to 1)	(-8 to -2)	(-5 to 0)	(-11 to -7)	(-8 to -5)			
Price	0.03	-0.03	-0.01	0.02	0.04	0.03	0.02	*Higher score is more healthful*		0.002^a^
	(0 to 0.05)	(-0.04 to -0.01)	(-0.04 to 0.01)	(0.01 to 0.05)	(-0.12 to 0.1)	(-0.06 to 0.08)	(-0.21 to 0.1)			
Promotions	0.5	0	-1	-1	0	-1	-1	*-5*	*7*	<0.001^a^
	(-1 to 1)	(-1 to 1)	(-3 to 0)	(-2 to -1)	(-0.4 to 0)	(-1 to 0)	(-1 to 0)			
Shelf placement	5	7	7	5	0	-3	-5	*-15*	*21*	<0.001^a^
	(4 to 7)	(5 to 9)	(4 to 9)	(4 to 7)	(-3 to 4)	(-6 to 3)	(-6 to 1)			
Store placement	4	4	4	4	1	-4	-4	*-15*	*21*	<0.001^a^
	(4 to 5)	(4 to 5)	(3 to 5)	(3 to 4)	(-4 to 5)	(-6 to 2)	(-6 to 1)			
Quality	17	17	17	17	5	5	0	*0*	*18*	<0.001^b^
	(17 to 17)	(16 to 18)	(15 to 18)	(15 to 17)	(0 to 10)	(0 to 13)	(0 to 8)			
Healthier alternative	4	5	2	3	0	1	1	*0*	*5*	<0.001^b^
	(4 to 5)	(5 to 5)	(2 to 3)	(2 to 3)	(0 to 1)	(1 to 2)	(0 to 2)			
Nutrition information	13	14	13	15	4	11	9	*0*	*15*	<0.001^b^
	(10 to 13)	(12 to 15)	(13 to 14)	(15 to 15)	(2 to 7)	(9 to 13)	(9 to 12)			
Single sale of two fruits^e^	100%	97%	26%	87%	23%	19%	21%	*0*	*2*	<0.001^c^

^a^Oneway analysis of variance, ^b^Kruskal-Wallis test, ^c^Chi square test, ^d^Median and inter-quartile range (IQR) were provided for both parametric and non-parametric variables for ease of reading, ^e^Percentage of two fruits available for single sale was provided because this variable was categorical, ^f^Possible range of scores for each variable except composite score which shows actual range of composite score values.

**Figure 3:**
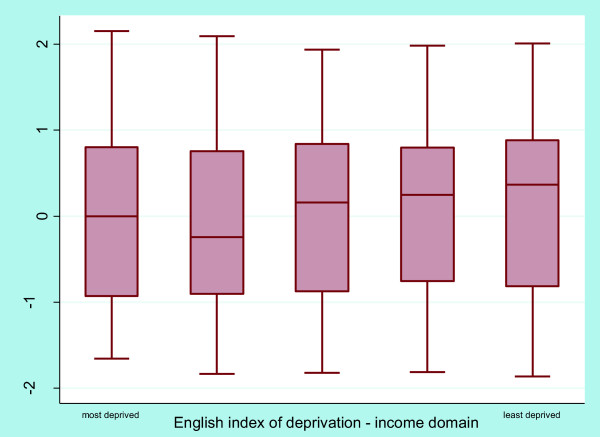
Box and whisker plot store healthfulness by level of neighbourhood deprivation.

Figure 3 shows a tendency towards store healthfulness improving with increasing levels of neighbourhood affluence. However, the test for trend revealed that this association was not significant (p = 0.09). Examination of the relationship between individual components of the healthfulness score with neighbourhood deprivation highlighted several disparities (Table 5). Fresh produce *quality* declined as level of neighbourhood deprivation increased (p < 0.01). The presence of *nutrition information* on less healthy items was greatest in the most affluent neighbourhoods while price *promotions* favoured less healthy products in all neighbourhoods except the most deprived (both p < 0.01). Prominent *shelf* and *store placement* of healthy products was slightly better in more affluent neighbourhoods (p = 0.04 and p = 0.05 respectively) and the availability of *healthier alternatives* of less healthy foods was worst in the most deprived neighbourhoods (p = 0.03). Product *variety, price* and *single fruit sale* were not associated with neighbourhood deprivation (all p ≥ 0.3).

**Table 5:** Individual variable and composite scores by neighbourhood deprivation

***Variable***	**Most deprived**	**2**	**3**	**4**	**Least deprived**	***Possible range***^***e***^		
			***Median (IQR)***^**c**^			***Min***	***Max***	***p-value***
**Composite score**	0	-0.2	0.2	0.2	0.4	*-1.9*	*2.2*	0.09^a^
	(-0.9 to 0.8)	(-0.9 to 0.8)	(-0.9 to 0.8)	(-0.7 to 0.8)	(-0.8 to 0.9)			
Variety	-7	-7	-7	-7	-7	*-25*	*35*	0.6^a^
	(-9 to -2.5)	(-10 to -2)	(-9 to -2)	(-8 to -4)	(-10 to -3)			
Price	0.02	0.03	0.02	0.03	0.02	*Higher score is more healthful*		0.5^a^
	(-0.04 to 0.06)	(-0.05 to 0.08)	(-0.04 to 0.06)	(-0.01 to 0.06)	(-0.01 to 0.06)			
Promotions	0	-1	-1	-1	-1	*-5*	*7*	<0.001^a^
	(-1 to 0)	(-1 to 0)	(-1 to 0)	(-2 to 0)	(-2 to 0)			
Shelf placement	2	0	3	3	4	*-15*	*21*	0.04^a^
	(-5 to 7)	(-5 to 5)	(-4 to 6)	(-3 to 6)	(-3 to 6)			
Store placement	1	0	2	2	2	*-15*	*21*	0.05^a^
	(-5 to 4)	(-5 to 4)	(-4 to 4)	(-4 to 4)	(-4 to 4)			
Quality	9	8	13	12	14	*0*	*18*	0.002^a^
	(1 to 15)	(0 to 15)	(3 to 17)	(0 to 17)	(3 to 17)			
Healthier alternative	1	2	2	2	2	*0*	*5*	0.03^a^
	(1 to 2)	(1 to 3)	(1 to 3)	(1 to 3)	(1 to 3)			
Nutrition information	12	12	12	12	13	*0*	*15*	0.003^a^
	(9 to 14)	(9 to 14)	(9 to 14)	(10 to 15)	(11 to 15)			
Single sale of two fruits^d^	42%	36%	39%	43%	43%	*0*	*2*	0.4^b^

^a^Spearman test for trend, ^b^Chi square test, ^c^Median and inter-quartile range (IQR) were provided for both parametric and non-parametric variables for ease of reading, ^d^Percentage of two fruits available for single sale was provided because this variable was categorical, ^e^Possible range of scores for each variable except composite score which shows actual range of composite score values.

## Discussion

The composite score of healthfulness varied across different types of retail food stores. Similar trends were observed for the scores of the nine component variables (variety, price, quality, promotions, shelf placement, store placement, nutrition information, healthier alternatives and single fruit sale) across the seven store types. These findings suggest that the composite score provided good representation of the individual components. Large and premium supermarkets consistently offered environments that supported more healthful food choices and small supermarkets generally offered healthful environments. Discount supermarkets, convenience, ‘world’ and petrol stores offered less healthful environments. However, these store types had widely spread scores, indicating that there were examples of better practice for each of these less healthful store types.

Level of neighbourhood deprivation did not predict store healthfulness. However, there was a trend towards affluent neighbourhoods having in-store environments more supportive of healthful food choices. The trends observed across the nine individual variable scores showed some disparities. Strong associations were observed for better quality produce and nutrition information in more affluent neighbourhoods, but unexpectedly, strong associations for healthier price promotion practices in the most deprived neighbourhoods. Weaker associations were found for poorer placement of healthy items and poorer availability of healthier alternatives in more disadvantaged neighbourhoods. No neighbourhood disparities were identified for product variety, price or single fruit sale. These results show how the composite score averages several multi-directional trends across the individual components. The healthfulness score also showed high internal (α = 0.86) [12] consistency which indicates that the components measure one internally consistent underlying construct.

Our finding that large and premium supermarkets offered environments that support consumers choosing healthful foods is consistent with previous research [11,37,38]. Unlike previous research [39-41], store size did not necessarily determine overall store healthfulness. Smaller versions of large supermarkets offered more healthful shopping environments than bigger discount supermarkets. Discount supermarkets offered the least supportive environment for healthy eating of all supermarkets in our study with the median composite score for discount supermarkets considerably (0.6 SD) lower than other supermarkets. Previous research in England has shown poorer quality and availability of healthy foods in discount supermarkets [42]. However, research from Scotland revealed better availability of healthy foods in discount stores and cheaper prices [43]. Our analysis of the component variables revealed that discount supermarkets had more varieties of less healthy than healthy products, promoted less healthy products more than healthy products and had fewer healthier alternatives of less healthy foods than other supermarkets. These findings suggest that discount supermarkets may be a site for future intervention, particularly for researchers or policy makers addressing health inequalities.

The healthfulness score we developed to measure overall in-store environment demonstrated the ability to discriminate within store types as well as between. For example, stores that are generally classified as ‘unhealthy’, such as petrol and convenience stores [44,45], revealed a spread in healthfulness scores from poor to good. This finding indicates there are examples of better, more healthful practices within less healthy store types and may also identify further subgroup categorisation of stores [46]. Further exploration of the specific differences and characteristics behind the better practices of more healthful stores may inform future store categorisation or identify targets for interventions to improve the environment of the least healthful food stores. Examples of successful initiatives to improve retail food stores exist [47], with interventions using multi-pronged strategies proving particularly effective [48]. Our composite measure of store healthfulness may provide a useful evaluation tool for future interventions addressing multiple in-store variables. In particular, the parametric nature of the healthfulness score provides more flexibility in statistical analyses than skewed measures.

Our results showed a trend for poorer store healthfulness in more deprived neighbourhoods however, this trend was not significant. Whilst no prior research has measured the full range of consumer nutrition environment factors included in this study, investigations in Scotland, England and Australia have shown little variation in availability and price by area level deprivation [49-51]. There are some illustrations in these countries for poorer fruit and vegetable quality [18,52] and greater promotion of less healthy products [21] in retailers located in more deprived areas however research is limited. In the US disparities in availability, price, variety and quality of healthy food exist and favour more affluent, predominantly white neighbourhoods [37,53]. More frequent prominent placement of less healthy items in poorer Latino areas compared with wealthier white areas has been observed but the findings were not significant [19]. Country differences may be due to the higher levels of urban residential segregation in the US than in countries such as Australia and the UK [54]. The geography of food retailing may also differ across countries. In the UK for example, over the last two decades major retailers have adopted an urban regeneration agenda locating large stores on the periphery of towns [55] as well as opening smaller stores in city and town centres [9,10]. These supermarket developments may have addressed some of the socioeconomic disparities in food access in the UK.

### Strengths and limitations

This study is the first to develop an overall measure of the consumer nutrition environment combining nine different variables into a single standardised score. The composite score characterised multiple environmental factors independent of measurement type; categorical, dichotomous and continuous measures were all represented equally in an overall score that was normally distributed. Using a standardised score provides a robust measure to conduct and interpret analyses and could ease examination of environmental and health/behavioural associations. The foods selected in this study are items which account for the most variance in tools used to discriminate between better and poorer dietary patterns in young women, young children and older adults. This selection could enable assessment of the relationship between environmental attributes of foods directly measured during dietary assessments in various populations in future research.

This study measured a much larger sample of stores than previous work [13,16,20] and covered 99% of retail food stores in the study area. This coverage provides a thorough representation of the variation of healthfulness of retail food stores and enhances confidence in the accuracy of the study results.

Good to excellent kappa statistics (0.73-0.95) were returned for almost all variables. These results are similar to other in-store audit tools (kappa >0.70) [56]. The sample of stores included in this reliability test however was small (n = 14) compared to similar studies (n = 30 to 85) [13,17,19]. The reliability (kappa 0.60) for fruit and vegetable quality was higher than the results reported in some studies [17], but lower than the results of others [57]. Future work using photos or a simple two-point scale of acceptable/unacceptable can provide a more consistent measure of quality as has been used in previous work [17,58]. Convergent validity against an alternative observational tool was not tested due to the intensive resources required for in-store audits. Data were collected from all store types and levels of neighbourhood deprivation over eleven months. Some aspects of seasonality or small price fluctuations may be been accounted for however, test-retest reliability and stability of items over time were not measured.

While our study was novel in assessing nine different in-store variables, three variables were restricted in their assessment. Shelf placement, store placement and nutrition information were all measured on the cheapest item available for each product category assessed. This restriction may have missed the opportunity to appropriately score the location of heavily promoted less healthy branded products, particularly in supermarkets which sell own-brand products. Other studies have assessed a specific brand and size of product [59] however, this approach is limited if not all stores stock that particular brand and size. For feasibility reasons specialty stores and restaurants were excluded. This study also has cross sectional and ecological limitations and the relationship between store healthfulness and diet was not assessed.

## Conclusion

This study used a large sample of retail food stores (n = 601) to develop a composite measure of the consumer nutrition environment incorporating nine different variables. The composite score showed good internal consistency and represented overall trends of the individual variables across seven different store types and five levels of neighbourhood deprivation. The composite measure showed differences between and within store types identifying opportunities for intervention in discount supermarkets and other store types with environments less supportive of healthful food choices. The standardised composite score developed in this study can offer greater flexibility in statistical analyses of environment-diet relationships in future observational and interventions studies than single in-store measures, which can be skewed. The products included in this tool relate to dietary quality measures, thus this tool can be used to examine relationships between environmental attributes of foods directly measured during dietary assessments in various populations in future research.

## Competing interests

Christina Black, Georgia Ntani, Hazel Inskip, Steve Cummins, Graham Moon and Janis Baird have no competing interests to declare and no financial disclosures to make.

Cyrus Cooper has no conflicts of interest to declare and has received consultancy, lecture fees and honoraria from AMGEN, GSK, Alliance for Better Bone Health, MSD, Eli Lilly, Pfizer, Novartis, Servier, Medtronic and Roche.

## Authors’ contributions

CB and JB conceived of the study, designed the audit tool, coordinated the data collection, healthfulness score development and analyses, and wrote the first draft of the manuscript. GN and HMI contributed to the development of the healthfulness score and performed the statistical analyses. CC, SC and GM participated in the design of the study and helped to draft the manuscript. All authors read and approved the manuscript.

## Supplementary Material

Additional file 1 Consumer nutrition environment audit tool and survey protocol. Click here for file
