# Supplementary material for: Measuring the healthfulness of food retail stores: variations by store type and neighbourhood deprivation
Source: Int J Behav Nutr Phys Act. 2014 May 23;11:69. doi: 10.1186/1479-5868-11-69 (PMC4132210; doi:10.1186/1479-5868-11-69)
Supplement: Additional file 1 — Consumer nutrition environment audit tool and survey protocol. [file 1479-5868-11-69-S1.docx]

Additional file 1. Consumer nutrition environment audit tool

Consumer nutrition environment audit tool protocol

**Consumer nutrition environment audit tool**

**Survey Instructions**

This tool has been developed to assess the availability, quality, cost, placement, nutrition information and promotion of selected food products identified from the 20-item Food Frequency Questionnaire (FFQ) in the Southampton Initiative for Health.[^1^](#_ENREF_1)^,^ [^2^](#_ENREF_2) The tool includes 12 products, six fruit and vegetables, and six shelf/cold products. To ensure consistency of data collection it is essential to follow this protocol closely.

Before you start

**Time:** All surveys are to be completed between 9am-6pm.

**ID badge:** Ensure your ID badge is clearly visible, clipped to your front or around your neck.

**Ask the manager:** Present the Nutrition, Exercise and Well-being Study Food Store Audit Information Sheet

to the store manager or supervisor. Briefly explain: *we investigating the dietary habits of women of*

*childbearing age and looking at how the food* *stores these women use relate to their dietary patterns. We are*

*approaching over 600 food stores* *across Hampshire. Could we complete a 10-15 minute survey of 12 food*

*products? The store or brand will* *not be individually identified.*

Front Page

**Complete the front page of the consumer nutrition audit tool:**

Interviewer: your initials

Date: DD/ MM/ YY

Geographical area: Southampton (S)/ Eastleigh (E)/ Fareham (F)/ Gosport (G)/ Havant (H)/ Portsmouth (P)

LSOA code: 4digit area code – see data collection schedule

Store ID: allocate chronologically as surveys are completed

Store type: See Table 1.

Opening Hours: Note times the store is open on each day of the week in 24 hour time. (24/7 = 08:00-08:00)

Number of cash registers: Note if there are **1, 2, 3, 4 OR 5+** cash registers that could be manned with a sales assistant. Exclude self-service cash registers, service desk and post office registers.

Start time & Finish time: Record the time (using 24 hour time) you start and the time you finish the survey. Use these measures to calculate the total time taken in minutes to complete the surveys in each store.

*Table 1. Store type descriptions*

| **Code** | **Store type** | **Description** | **Examples** |
| --- | --- | --- | --- |
| 0 | Premium supermarket | 5+ manned cash registers  Promoted as offering highest quality goods and service | Waitrose, M&S |
| 1 | Large supermarket | 5+ manned cash registers  All foods & many varieties  Majority of supermarket share | Tesco, Sainsburys, Asda, Morrisons |
| 2 | Discount supermarket | 5+ manned cash registers  Heavily promoted as low price stores | Aldi, Lidl, Iceland, Netto, Kwiksave |
| 3 | Small supermarket | 1-4 manned cash registers  Smaller store of known brand name | Tesco Express, Co-Op, Sainsburys Local |
| 4 | ‘World’ store | 1-4 manned cash registers  Products for specific ethnicities | Asian supermarkets, Polish supermarkets, World Foods |
| 5 | Convenience store | 1-4 manned cash registers  Limited number of products  Independents & ‘symbols’^b^ | Spar, OneStop, MACE, Independent stores |
| 6 | Petrol station store | Sell petrol/diesel  Includes small supermarkets that sell petrol | Shell Select, Tesco Petrol Station, BP, M&S |

^b^ ‘Symbol’ convenience stores are affiliated with a symbol group brand such as OneStop and Spar.

Survey Variables – **ONLY include products that are available for purchase**

1. **Number of varieties:** Variety encompasses product flavour, product size, ‘fair trade’/’organic’ product and budget product. The number of varieties however is specific to each product (details below). The number of varieties is categorised using: **1, 2, 3, 4, 5+ OR 9** (none available)
2. **Quality:** **Vegetable and Fruit products ONLY**

Quality assessment is based on the condition of the vast majority of the proxy product (details in Table 2). If the produce in brackets is not available please assess the quality of the cheapest variety of the product. The quality is categorised using:

| 3 | High/ Good |
| --- | --- |
| 2 | Medium |
| 1 | Low/ Poor |

1. **Healthy Option: Shelf, Cold and Frozen products ONLY**

A healthier option is a product that has a nutrient claim on the front of the pack indicating it is lower in total fat, sugar or salt AND/OR is higher in a nutrient intrinsically part of the product (details below).

Note Y (yes) or N (no) if ≤1 healthier option is available.

1. **Product price:** Note the price (£0.00) of the cheapest product.

Note the sale price if the product is on special offer but exclude BOGOFs where 2 items must be purchased.

1. **Product weight:** Note the product weight in grams clearly in the space provided.

If the weight of a vegetable product is not identified apply standardised weights in Table 3.

Note the number of tablets instead of weight for artificial sweetener tablets.

*Table 3. Standard product weights*[*^3^*](#_ENREF_3)

| **Product** | **Description** | **Weight** |
| --- | --- | --- |
| Peppers | 1 medium red/green | 160g |
| Tomatoes | 1 small tomato  1 medium tomato  1 large/beefsteak tomato  1 cherry tomato | 65g  85g  150g  15g |
| Lettuce | 1 iceberg  1 round/ 2x little gem  2 medium/ 1 large Romaine flute | 250g*  94g*  180g* |
| Onions | 1 small brown onion  1 medium brown/red  1 large brown | 60g*  90g  120g* |
| Apples | 1 small apple  1 medium apple  1 large apple | 75g  112g  170g |
| Bananas | 1 small banana without skin  1 medium banana without skin  1 large banana without skin | 80g  100g  120g |
| Bread | Small sliced loaf  Large sliced loaf | 400g  800g |

*Table 4. Other useful weights*

| **Description** | **Weight** |
| --- | --- |
| 1 pound | 454g |
| 2 pounds | 907g |
| 1 kilo | 1000g |
| 4 kilos | 4000g |

1. **Product description:** Note the brand and name of the product.

Also note the packaging description if the weight of the product is not available or if the product is a multi-pack.

For example write 3 mixed peppers or 6 x 25g for a crisps multi-pack.

1. **Placement - shelf:** Note the number representing the shelf placement of the cheapest item and cheapest healthier option of the product category.

Shelf placement is categorised using the 3 scales below. Prominent = eye level/ obvious placement. The diagram below provides guidance on using the descriptors.

If the product is placed on more than one shelf (ie 2 & 3) score the product as the highest possibility (3).

| 3 | Prominent |  |  |  |
| --- | --- | --- | --- | --- |
| 2 | Other |  |  |  |
| 1 | Bottom |  |  |  |
|  |  |  |  |  |
| Prominent |  |  |  |  |
| Prominent | Prominent | Prominent |  |  |
|  |  |  | Prominent |  |
| bottom | bottom | bottom | bottom | bottom |

**5 shelves 4 shelves 3 shelves 2 shelves 1 shelf**

1. **Placement - store:** Please note the number representing the store placement of the cheapest item and cheapest healthier option of the product. Product placement in the store is categorised using:

| 3 | Prominent | I see the product when I get to/enter the store  OR it’s at the end of an aisle |
| --- | --- | --- |
| 2 | Noticeable | When I go looking I see the product |
| 1 | Inconspicuous | After I have been looking for a while I see the product |

1. **Nutrition information:** Please note the number representing the nutrition information on the cheapest item and cheapest healthier option. Nutrition information must be easily visible before purchase.

| 3 | Front & back of pack | Front of pack = full GDA or traffic light labelling (below) |
| --- | --- | --- |
| 2 | Back of pack |  |
| 1 | Other | Please note the type of information ie. recipe card |
| 0 | None |  |

GDA labelling
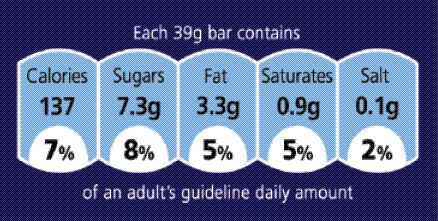
 Traffic light labelling
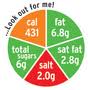
 &
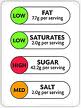


OR

An obvious nutrition table on the front of the product pack
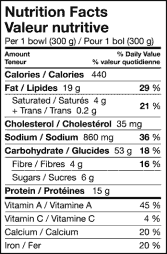


Note the front of pack for bread includes the sides and ends because of how bread is displayed on the shelf.

1. **Promotion:** Please note Yes or No if there is promotion on **any** product within the product category. Exclude reduced price items that may have poorer quality or shorter shelf life.

Process to create a composite score of healthfulness for each store – **to be used following data collection, data entry and data cleaning.** Statistical expertise is required.

Peppers

**Variety**

| **Definition of Variety** | **Coding Tips** |  |
| --- | --- | --- |
| - Different colours  - Different size/pointed shape  - Organic/fair-trade  - Economy Range | Each colour counts as a different variety – except multipacks = 1 variety  Size & shape only count if the pepper is a different species (different name)  Organic & fair-trade products = 1 variety even if both are available  One or more economy products = 1 variety | Exclude multipacks as a variety when colour is repeated  Only count organic/fair-trade when an additional choice  Only count economy when an additional choice |

**Quality**

| **Product** | **Coding options** | | |
| --- | --- | --- | --- |
|  | **High/ Good (3)** | **Medium (2)** | **Low/ Poor (1)** |
| **Loose red peppers**  (Cheapest peppers if loose red pepper are not available) | Good strong intense **bright red** colour, uniform shape and size, **free from cracks**, decay, mould and fungi, clean no blemishes, bruises or marks, firm crisp product, **tight skin** | Good red colour, mixed sizes, **occasional blemish**, no bruises or soft marks, firm product | Soft product, **brown marks**, blemishes, moulds, **wrinkled/ wilted skin**, product drying out |

**Standard Price -** Note price **per Kg or 100g** **or per piece** for **cheapest** **loose** and **cheapest** **packed** items AND the **name/colour** and how the items are packed

Tomatoes

**Variety**

| **Definition of Variety** | **Coding Tips** | **Exclusions** |
| --- | --- | --- |
| - Different species  - Different size/shape  - Organic/fair-trade  - Economy Range | Each species (cherry, beef, plum etc) counts as a different variety  Size & shape only count if tomatoes are a completely new species  Organic & fair-trade products = 1 variety even if both are available  One or more economy products = 1 variety | Vine-ripened tomatoes are not considered a new variety  Apply the first name of a product where it is a cross-species ie. cherry plum is classed as in cherry variety  Only count organic/fair-trade when an additional choice  Only count economy when an additional choice |

**Quality**

| **Product** | **High/ Good (3)** | **Medium (2)** | **Low/ Poor (1)** |
| --- | --- | --- | --- |
| **Loose medium**  (cheapest) | **Bright green/red colour**, consistently sized products, **no blemishes**, bruises or abrasions, firm turgid product, **shiny skin** | Shiny red colour (90%) for variety, no bruising, slight size variation, **firm product**, **occasional blemish**, perhaps a few marks, no bruises | **Dull red colour**, marks, blemishes, bruised, product feels soft, skin not turgid but **wrinkled/wilted** |

**Standard Price -** Note price **per Kg or 100g** **or per piece** for **cheapest** **loose** and **cheapest** **packed** items AND the **name** and how the items are packed

Lettuce

**Variety**

| **Definition of Variety** | **Coding Tips** |  |
| --- | --- | --- |
| - Different species  - Bagged lettuce  - Organic/fair-trade  - Economy Range | Different sizes of the same species = 1 variety (gem hearts & normal gem)  Bagged washed lettuce = 1 variety (no matter how many different types)  Organic & fair-trade products = 1 variety even if both are available  One or more economy products = 1 variety | Only count organic/fair-trade when an additional choice  Only count economy when an additional choice |

**Quality**

| **Product** | **Coding options** | | |
| --- | --- | --- | --- |
|  | **High/ Good (3)** | **Medium (2)** | **Low/ Poor (1)** |
| **Iceberg**  (Cheapest lettuce if iceberg is not available) | Good **bright green colour**, round head, **crisp** turgid feel/**appearance**, clean, **no** blemishes, or **browning of leaves** | **Light green colour**, no browning, **occasional blemish** or dirt | Weak colour, **product looks flaccid**, leaves not firm, water loss, **browning on leaves**, soil and mud on product, evidence of slugs, **insect damage**, spotting, brown stain |

**Standard Price -** Note price **per Kg or 100g** **or per piece** for **cheapest** **loose** and **cheapest** **packed** items AND the **name** and how the items are packed. Please note that a singly wrapped iceberg lettuce is considered a loose item.

Onion

**Variety**

| **Definition of Variety** | **Coding Tips** |  |
| --- | --- | --- |
| - Different species  - Different sizes/ colour  - Organic/fair-trade  - Economy Range | Each species counts as a different variety (red, large mild brown, shallots etc)  Size & colour only count if the onion a different species (different name)  Organic & fair-trade products = 1 variety even if both are available  One or more economy products = 1 variety | Spring onions are not included  Pickled onion are not included  Only count organic/fair-trade when an additional choice  Only count economy when an additional choice |

**Quality**

| **Product** | **Coding options** | | |
| --- | --- | --- | --- |
|  | **High/ Good (3)** | **Medium (2)** | **Low/ Poor (1)** |
| **Loose Medium Brown**  (Cheapest onion if not available) | Bright, good colour, **no blemishes**, bruises or marks, firm product, **skin intact** | **Occasional blemish**, perhaps a few marks, no bruises, **firm to touch** | Marks, **blemishes/moulds**, bruised, brown/black blotches, defects, greening of fleshy scales, **leathery skin**, soft to touch, product dried out |

**Standard Price -** Note price **per Kg or 100g** **or per piece** for **cheapest** **loose** and **cheapest** **packed** items AND the **name** and how the items are packed

Apples

**Variety**

| **Definition of Variety** | **Coding Tips** |  |
| --- | --- | --- |
| - Different species/ colours  - Bagged sliced apples  - Organic/fair-trade  - Economy Range | Species identified in name = 1 different variety  Bagged sliced apples = 1 variety (no matter how many different types)  Organic & fair-trade products = 1 variety even if both are available  One or more economy products = 1 variety | Only count organic/fair-trade when an additional choice  Only count economy when an additional choice |

**Quality**

| **Product** | **Coding options** | | |
| --- | --- | --- | --- |
|  | **High/ Good (3)** | **Medium (2)** | **Low/ Poor (1)** |
| **Cheapest loose eating apples**  (Cheapest packed apples if loose apples are not available) | Good strong intense **bright** red/green **colour**, **no blemishes**, bruises or marks, firm product, **tight skin** | Good red/green colour for variety, **occasional blemish**, perhaps a few marks, **no bruises**, **firm product**, looks good | **Weak** red/green **colour**, marks, **blemishes**, bruised, blackened, soft, **wrinkled/wilted skin**, looks like it should be eaten immediately |

**Standard Price -** Note price **per Kg or 100g** **or per piece** for **cheapest** **loose** and **cheapest** **packed** items AND the **name** and how the items are packed

Bananas

**Variety**

| **Definition of Variety** | **Coding Tips** | **Exclusions** |
| --- | --- | --- |
| - Different species  - Different size  - Organic/fair-trade  - Economy Range | Species identified in name = a different variety  If size is specified in the name = 1 variety (ie kids/ lunchbox bananas)  Organic & fair-trade products = 1 variety even if both are available  One or more economy products = 1 variety | Ripen at home is not an additional variety  Only count organic/fair-trade when an additional choice  Only count economy when an additional choice |

**Quality**

| **Product** | **High/ Good (3)** | **Medium (2)** | **Low/ Poor (1)** |
| --- | --- | --- | --- |
| **Loose medium**  (cheapest) | **Strong green/yellow colour**, **no black marks**, blemishes or bruises, **product firm** | Predominately yellow/green stalk, occasional blemish, **perhaps a few marks**, no bruises, **firm product**, looks good | **Brown marks on skin**, blackening, wizened stalk, other blemishes, **product feels soft**, looks like it **should be eaten immediately or use for cooking** |

**Standard Price -** Note price **per Kg or 100g** **or per piece** for **cheapest** **loose** and **cheapest** **packed** items AND the **name** and how the items are packed

Oven chips

**Variety**

| **Definition of Variety** | **Coding Tips** | **Exclusions** |
| --- | --- | --- |
| - Different types  - Different sizes  - Healthier options  - Organic/fair-trade  - Economy Range | Types included – Smilies, Wedges, microwaveable  Different sizes = different varieties – (eg. jumbo, normal, French fries)  One or more healthier options = 1 variety  Organic & fair-trade products = 1 variety even if both are available  One or more economy products = 1 variety | Frozen frying chips are not included  Different cuts (crinkle/straight) are not different varieties  Only count organic/fair-trade when an additional choice  Only count economy when an additional choice |

**Healthier Option**

| **Nutrient claims included** | **Example** |
| --- | --- |
| Lower total fat  Lower salt | *Less 4% total fat*  *20% less salt than regular oven chips* |

**Standard Price -** Note price **per Kg or 100g** **or per piece** for **cheapest** **regular** and **cheapest healthier** items AND note item **name, brand** & how the item is packed

Sausages

**Variety**

| **Definition of Variety** | **Coding Tips** | **Exclusions** |
| --- | --- | --- |
| - Different flavours  - Different sizes  - Microwavable  - Healthier options  - Organic/fair-trade  - Economy Range | Count different flavours all fresh & frozen sausages first  One or more chipolatas/ smaller sausages = 1 variety  One or more microwavable sausage = 1 variety  One or more healthier options = 1 variety  Organic & fair-trade products = 1 variety even if both are available  One or more economy products = 1 variety | Cooked sausages are not included  Sausage rolls are not included  Party sausages, salami etc are not included  Vegie sausages are not included  Only count organic/fair-trade when an additional choice  Only count economy when an additional choice |

**Healthier Option**

| **Nutrient claims included** | **Example** |
| --- | --- |
| Lower total fat  Lower salt | *30% less fat than regular sausages*  *20% less salt than regular sausages* |

**Standard Price -** Note price **per Kg or 100g** **or per piece** for **cheapest** **regular** and **cheapest healthier** items AND note item **name, brand** & how the item is packed. Note if the cheapest sausages (regular and healthy option) are cold or frozen.

Sugar

**Variety**

| **Definition of Variety** | **Coding Tips** | **Exclusions** |
| --- | --- | --- |
| - Different types  - Healthier options  - Organic/fair-trade  - Economy Range | Different types are different varieties (eg. white, icing, caster, Demerara)  One or more healthier options = 1 variety  Organic & fair-trade products = 1 variety even if both are available  One or more economy products = 1 variety | Exclude icing sugar  Only count organic/fair-trade when an additional choice  Only count economy when an additional choice |

**Healthier Option**

| **Nutrient claims included** | **Example** |
| --- | --- |
| Artificial sweeteners – (virtually no calories) | *Only 0.2 calories per tablet*  *Low calorie sweetener* |

**Standard Price -** Note price **per Kg or 100g** **or per piece** for **cheapest** **regular** and **cheapest healthier** (granulated & tablets) items AND note item **name, brand** & how the item is packed

Crisps

**Variety – all potato crisps**

| **Definition of Variety** | **Coding Tips** | **Exclusions** |
| --- | --- | --- |
| - Different flavours  - Different sizes  - Healthier options  - Organic/fair-trade  - Economy Range | Count different flavours (cheese & onion, ready salted etc) first  Each different size other than the main size available = 1 variety  One or more healthier options = 1 variety  Organic & fair-trade products = 1 variety even if both are available  One or more economy products = 1 variety | Rice, vegetable, wheat, corn and prawn/oriental crackers are not included  Only count organic/fair-trade when an additional choice  Only count economy when an additional choice |

**Healthier Option – only salted potato crisps**

| **Nutrient claims included** | **Example** |
| --- | --- |
| Lower total fat  Lower salt | *30% less fat than regular salted crisps*  *20% less salt than regular salted crisps* |

**Note the price and availability if a healthier option of crisps is available in a flavour other than plain salted. Please note the flavour.**

**Standard Price -** Note price **per Kg or 100g** **or per piece** for **cheapest** **regular** and **cheapest healthier** items AND note item **name, brand** & how the item is packed

Wholemeal bread – sliced bread only

**Variety**

| **Definition of Variety** | **Coding Tips** | **Exclusions** |
| --- | --- | --- |
| - Different types  - Loaf size  - Fortification  - Healthier options  - Organic/fair-trade  - Economy Range | Different types of sliced brown bread (eg. seed & oats, rye, wholemeal etc )  Availability of one or more half loaves = 1 variety  Omega 3/ calcium fortification = 1 variety  One or more healthier options = 1 variety  Organic & fair-trade products = 1 variety even if both are available  One or more economy products = 1 variety | Only count breads that are in the bread section (do not go searching for gluten free breads etc)  Different cuts (thick/ medium) are not different varieties  Only count organic/fair-trade when an additional choice  Only count economy when an additional choice |

**Healthier Option**

| **Nutrient claims included** | **Example & notes** |
| --- | --- |
| Lower salt  Lower sugar | *20% less salt than regular wholemeal bread* |

**Standard Price -** Note price **per Kg or 100g** **or per piece** for **cheapest** **regular** and **cheapest healthier** items AND note item **name, brand** & how the item is packed

White bread – sliced bread only

**Variety**

| **Definition of Variety** | **Coding Tips** | **Exclusions** |
| --- | --- | --- |
| - Different types  - Half loaf  - Fortification  - Healthier options  - Organic/fair-trade  - Economy Range | Different types of sliced white bread (eg. farmhouse, white)  Availability of one or more half loaves = 1 variety  Omega 3/ calcium fortification = 1 variety  One or more healthier options = 1 variety  Organic & fair-trade products = 1 variety even if both are available  One or more economy products = 1 variety | Only count breads that are in the bread section (do not go searching for gluten free breads etc)  Different cuts (thick/ medium) are not different varieties  Crusts off is not a different variety  Only count organic/fair-trade when an additional choice  Only count economy when an additional choice |

**Healthier Option**

| **Nutrient claims included** | **Example & notes** |
| --- | --- |
| Lower salt  Lower sugar  Higher fibre | *20% less salt than regular white bread*  *50% more fibre than regular white bread*  *Counts as one of your daily serves of wholegrain*  Fortification in calcium/ omega 3 etc is NOT a healthier option |

**Standard Price -** Note price **per Kg or 100g** **or per piece** for **cheapest** **regular** and **cheapest healthier** items AND note item **name, brand** & how the item is packed
